# A method for designing birdcage coils based on a simplified magnetic field model, validated experimentally at 4 T, 7 T, and 15.2 T

**DOI:** 10.1016/j.ohx.2025.e00729

**Published:** 2025-12-13

**Authors:** A. Villarreal, J. Lazovic, S.E. Solis-Najera, R. Martin, R. Ruiz, L. Medina, A.O. Rodriguez

**Affiliations:** a Departamento de Fisica, Facultad de Ciencias, UNAM, Mexico City 04510, Mexico; b Department of Physical Intelligence, Max Planck Institute for Intelligent Systems, Stuttgart 70569, Germany; c Department of Electrical Engineering, UAM, Iztapalapa, Mexico City 09340, Mexico

**Keywords:** Magnetic resonance imaging, RF volume coil, Birdcage resonator, Theoretical magnetic field, Ultra high field, Preclinical hardware

## Abstract

Magnetic resonance imaging and spectroscopy rely on magnetic fields generated by radiofrequency (RF) volume coils for high-quality data acquisition. Understanding electromagnetic field behavior in these coils is key to optimizing imaging and designing advanced coils. This paper presents an alternative tool to validate experimental and simulated results from traditional bridge coils using a theoretical approach to derive the magnetic field B1 expression for a BC coil. The formula will be used for: (a) assessing RF coil performance for high-quality images with optimal SNR and accurate anatomical representation, and (b) guiding the development of BC coils for specific applications. To validate the model, phantom images were acquired at different resonant frequencies, and the results were compared with experimental data. The findings confirm the accuracy and effectiveness of the model, offering insights into electromagnetic field behavior and providing a framework for advancing RF coil design in MR imaging and MR spectroscopy.

## Graphical abstract

**Figure:**
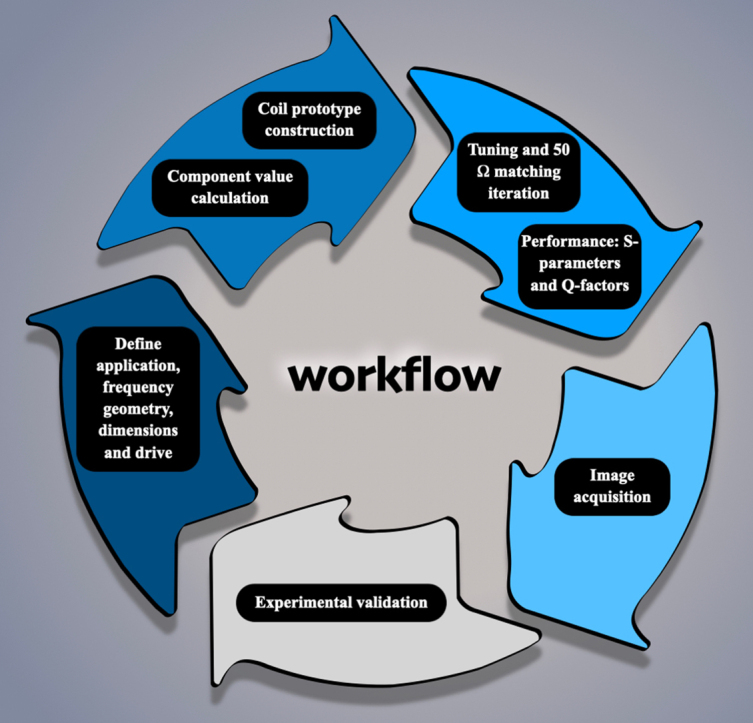

## Specifications table

**Table:** 

Hardware name	Birdcage coil for high and ultra high field at 4 T, 7 T and 15.2 T
Subject area	Magnetic Resonance Imaging, Biomedical Engineering
Hardware type	Imaging devices
Closest commercial analog	Commercial birdcage coils
Open source license	Creative Commons Attribution-ShareAlike 4.0 International License (CC BY-SA 4.0)
Cost of hardware	551 USD
Source file repository	10.5281/zenodo.14963089

## 1 Hardware in context

Different types of RF coils are employed in MRI systems, depending on the specific requirements of the scan, such as coils designed for particular body regions or specialized imaging techniques. RF coils are essential components of the MRI acquisition, as they enable the generation of high-resolution images that are critical for diagnosing and evaluating a wide range of medical conditions [1]. Key factors such as coil sensitivity and uniformity play a crucial role in the performance of these devices, particularly when designing coils for diverse applications. Among the various RF coil designs, birdcage (BC) coils are particularly favored in MRI due to their ability to provide a high signal-to-noise ratio and excellent homogeneity of the RF magnetic field [2]. These characteristics ensure a large, uniform field of view, making BC coils ideal for imaging large anatomical regions or for applications requiring high spatial resolution. Despite advances in coil technology over the years, BC coils remain a primary choice for both preclinical and clinical MRI applications due to their reliability, versatility, and superior imaging performance [3], [4], [5].

Developing RF coils for MRI typically requires specialized software, which often comes with high licensing costs. These tools, such as CST Studio, COMSOL Multiphysics, and Ansys HFSS, are used for electromagnetic simulations, coil design, and optimization. They help model and simulate the electromagnetic properties of RF coils, ensuring they meet the required design and performance standards for MRI applications. Furthermore, these software tools demand significant computational power, particularly when simulating complex 3D geometries, varying material properties, and the interaction between the RF coil and the magnetic field. This makes simulations time-consuming, often requiring high-performance computing resources like powerful workstations or access to supercomputing clusters.

In this paper, we present an alternative and independent tool to validate both experimental and simulated results obtained with the traditional bridge coil. We employ a classic theoretical approach to derive an expression for the magnetic field B1 generated by a BC coil. The derived formula will be used for: (a) assessing RF coil performance to acquire high-quality images with optimal signal-to-noise ratios (SNR) and accurate anatomical representation, and (b) guiding the development of BC coils designed to meet the specific needs of various applications.

Our method is based on the expansion of first- and second-order spherical Bessel functions, which provides an efficient means of characterizing the electromagnetic field within the BC coil. This approach simplifies the mathematical complexity typically associated with the analysis of RF coils, making it easier to predict the spatial distribution of the magnetic field. To validate the accuracy and effectiveness of the derived expression, we conducted a series of experiments involving the acquisition of phantom images using both in-house and vendor-provided BC coils across multiple resonant frequencies. We then compared the experimental data obtained from these measurements with the theoretical results derived from our magnetic field expression.

## 2 Hardware description

The study utilized both vendor-provided and in-house BC coils, ensuring a comprehensive evaluation of performance and design variations between commercially available and custom-built models.

**(a) High-Pass Birdcage Coil at 300 MHz:**

A high-pass birdcage coil with a diameter of 4 cm and a length of 6.4 cm was designed with four rungs to achieve a low specific absorption rate (SAR), as detailed in [6]. Strips were mounted and distributed equidistantly around the perimeter of an acrylic cylinder, and two circular end rings joined the structure. Fixed-value chip capacitors (American Technical Ceramics, series ATC 100 B nonmagnetic) of 8.8 pF and 7.8 pF were soldered into the two conducting end rings to generate resonant modes for the birdcage and rectangular slot coils, respectively. A tuning and matching network for each channel, along with BNC-type coaxial connectors, were also soldered to both end rings. A 50 Ω matching and fine-tuning system was achieved using two nonmagnetic trimmers (Voltronics, Corp: 1–33 pF, NMAJ30 0736), with one trimmer attached to each channel. The transceiver coil prototype was quadrature-driven for improved performance. With a diameter-to-length ratio of 0.625, the coil was optimized for enhanced field homogeneity and minimized signal-to-noise ratio (SNR) degradation. Operating in quadrature mode and in transceiver configuration, the coil demonstrated efficient performance, making it well-suited for the intended application.

**(b) Low-Pass Birdcage Coil at 300 MHz:**

This low-pass birdcage coil [7] was carefully constructed using high-quality copper strips for the four rungs, mounted symmetrically along the perimeter of a durable acrylic cylinder. The ends were secured with precision-machined circular end rings. Resonance was achieved through fixed-value, non-magnetic chip capacitors (American Technical Ceramics, series ATC 100 B, 8.8 pF and 7.8 pF), which were soldered onto the end rings. These capacitors enabled stable resonant modes for both birdcage and rectangular slot coil configurations. A well-designed tuning and matching network was integrated, with BNC connectors for seamless signal transmission. For precise 50 Ω matching and fine-tuning, two non-magnetic trimmers (Voltronics Corp: 1–33 pF, NMAJ30 0736) were added, one for each channel. The quadrature-driven transceiver coil delivered improved signal uniformity, reduced artifacts, and enhanced performance, making it ideal for high-resolution imaging and other advanced MR applications.

**(c) BC Coil for 300 g Rats at 300 MHz:**

The high-pass birdcage coil [8] for use with rats weighing approximately 300 g was designed with 16 rungs to achieve optimal $B_{1}$ field uniformity, as recommended by Doty et al. [9]. Copper strips were evenly distributed around the perimeter of an acrylic cylinder, with the ends connected by two circular copper end rings. Non-magnetic chip capacitors (American Technical Ceramics, series ATC 100 B, 18 pF) were soldered to the end rings to facilitate resonance. A comprehensive tuning and matching network was implemented, with BNC connectors at both ends. Fine-tuning was achieved using two non-magnetic 1–33 pF variable capacitors (Voltronics Corp, NMAJ30 0736) for each channel. To further enhance performance, cable traps were incorporated between the BNC connectors and matching capacitors. These cable traps, constructed from coiled coaxial cable with eight turns and a 1.5 cm inner diameter, were equipped with variable capacitors to adjust resonance to the 300 MHz Larmor frequency. The quadrature-driven transceiver coil optimized signal uniformity, reducing artifacts and ensuring high-quality MR imaging.

**(d) Vendor-Provided Birdcage Coil for Comparison at 300 MHz:**

For comparison, a vendor-provided quadrature birdcage coil operated in transceiver mode was used [10]. This coil had similar dimensions and an equal number of rungs to the custom-designed models. It was the RF RES 300 1H 075/040 QSN TR (model no.: 1PT13161V3, serial no.: S0121, REV/VEC: 2P01.05, Bruker BioSpin MRI, GmbH, Germany) and provided performance benchmarks against the custom prototypes.

**(e) Birdcage Coil at 170 MHz:**

A pass-band birdcage coil was developed for operation at 170 MHz, as shown in Fig. 2e. This coil, designed with an optimized configuration based on [11], had a length of 12 cm and an inner diameter of 18 cm. The 4-leg architecture utilized 2 cm wide copper strips for the legs, and the circular end rings had a diameter of 18 cm. For quadrature operation, two 50 Ω coaxial cables were connected to the coil. Non-magnetic capacitors (23.2 pF and 12 pF) were symmetrically distributed around the coil’s circumference. Matching was achieved using two non-magnetic 30 pF variable capacitors for fine-tuning to 170.29 MHz. The 4 cm spacing between the copper strips minimized mutual inductance effects, ensuring high-performance operation. This meticulously engineered design delivered stable and efficient MR imaging, optimized for precise field homogeneity and minimal interference.

**(f) Birdcage Coil at 650 MHz:**

A quadrature transceiver birdcage coil [12] was employed for 650 MHz operation at 15.2 T (Bruker Co, Ettlingen, Germany, model RES 650 1H 059/035 QSN TR), with an inner diameter of 35 mm, outer diameter of 6 cm, and a length of 3 cm. This high-frequency coil was used for advanced MR imaging, offering excellent performance in high-field applications.

**Fig. 1:**
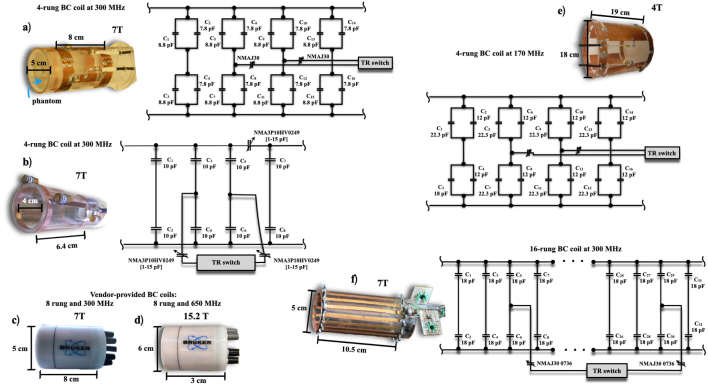
Photographs of the birdcage coils studied. Panels (a, b, e, f) depict the in-house built coils alongside their equivalent circuits, while panels (c, d) show the vendor-provided coil. Key dimensions are highlighted for comparison.

Fig. 1 presents photographs showcasing all the BC coils, providing a visual comparison of their designs, construction details and their corresponding equivalent circuits.

## 3 Design files summary

**Shield External Base**

A cover with an internal diameter of 3.3 cm, an external diameter of 6.620 cm, and a height of 13.1 cm, a thickness of 8 mm was meticulously designed and 3D printed using black 0.175 cm PLA filament from the Color Plus brand. This component is an integral part of the final design, as illustrated in the assembly diagram.

**Birdcage coil support**

A base with an internal diameter of 3.3 mm, an external diameter of 6.62 cm, and a thickness of 0.8 cm was designed, incorporating two 0.51 cm holes for cable routing, and a height of 12.16 cm. It was 3D printed using black 0.175 cm PLA filament from the Color Plus brand. This component is an essential part of the final design, as shown in the assembly.

**Birdcage coil 1**

The external cover of the antenna was designed with an internal diameter of 63 mm, an external diameter of 6.62 cm, a thickness of 0.16 cm. It was 3D printed using black 1.75 mm PLA filament from the Color Plus brand. This component is an integral part of the final design, as depicted in the assembly.

**Birdcage coil 2**

The antenna support was designed with an internal diameter of 33 mm, an external diameter of 40 mm, a thickness of 3.50 mm, and a height of 130 mm. It was 3D printed using black 1.75 mm PLA filament from the Color Plus brand. This component is an integral part of the final design, as shown in the assembly.

**Final Assembly of the Birdcage**

**Fig. 2:**
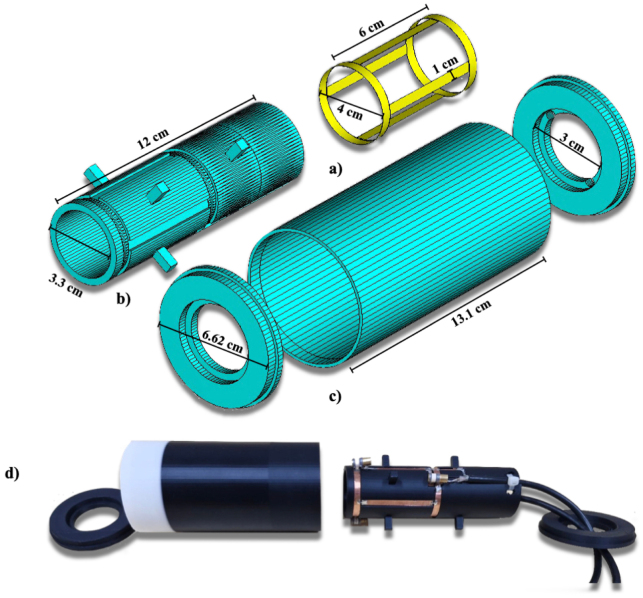
Components of a 4-rung birdcage coil. (a) Complete coil assembly. (b) Structural support. (c) Protective cover. (d) Photograph of the final constructed coil. All renderings include relevant dimensions.

The final assembly integrates all designed components, ensuring precise alignment and optimal functionality. Each part was 3D printed using black 1.75 mm PLA filament from the Color Plus brand and carefully assembled to form the complete structure, as depicted in the final design. To illustrate the construction of a birdcage coil, we outline the general procedure for a 4-rung design used in preclinical MRI in Fig. 2 (see Table 2).

## 4 Bill of materials summary

**Table 1:** Catalogue of engineering design files.

Design filename	File type	Open source license	Location of the file
Shield external base	CAD file	Creative Commons for Open Source Hardware (COSH)	Zenodo Link
Final assembly of the birdcage	CAD file	Creative Commons for Open Source Hardware (COSH)	Zenodo Link
Birdcage coil support	CAD file	Creative Commons for Open Source Hardware (COSH)	Zenodo Link
Custom-built birdcage coil 1	CAD file	Creative Commons for Open Source Hardware (COSH)	Zenodo Link
Custom-built birdcage coil 2	CAD file	Creative Commons for Open Source Hardware (COSH)	Zenodo Link

**Table 2:** Inventory of constituent components.

Designator/name	Number	Cost/unit [US dollars]	Total cost	Source of material	Material type
Copper sheet	1	4/1 m	4	Aceros y Metales Cuautitlan (acerosymetalescuautitlan.com.mx)	Copper

Self adhesive copper tape 25.4 mm	2	20/1	40	3M Mexico (www.3m.com.mx)	Copper

Ceramic capacitor	10	6.5/1	65	Mouser Electronics (www.mouser.com)	Semiconductor

Coaxial cable (50 Ohm impedance)	10 m	5/1 m	50	PCdigital (www.pcdigital.com.mx)	Silver coated metal

BNC (RF - 50 Ohm) male/female	10	11/1	110	L-com (www.l-com.com)	Lead

Wire solder	2	31.25/1	62.50	FCTA Mexico (fctamexico.mx)	Lead

Acrylic materials	5	15/1 tube	30	AvanceyTec (avanceytec.com.mx)	Acrylic

Ceramic trimmers (1.5 pF - 30 pF)	6	30	180	DigiKey (www.digikey.com.mx)	Polytetrafluoroethylene (PTFE)

Plastic C-clamps	5	4.51/1	9	Olson (clampsmexico.com)	Plastic material

Total cost			550.5		

## 5 Build instructions

All in-house BC coil prototypes were constructed following a method similar to that described by Kemper et al. [13] for birdcage coil design. However, instead of utilizing a 3D printer, we opted for manual fabrication of the rungs and end rings by cutting them from copper sheets. These copper components were carefully shaped and then assembled onto acrylic cylinders to form the complete birdcage coil structure. To ensure proper functionality, both non-magnetic chip capacitors and trimmers were soldered onto the coil to form high-pass and low-pass versions of the BC coil, depending on the specific design requirements.

The values of the passive components, including capacitors and trimmers, were initially estimated using the OpenBirdcageBuilder [14] software, a tool specifically developed to assist in the design and optimization of birdcage coils. This allowed us to obtain a preliminary set of values for the components needed to achieve the target resonant frequencies. After component selection, fine-tuning and 50 Ω impedance matching were performed using a network analyzer to precisely adjust the coil’s resonant frequency and impedance characteristics.

The process of tuning involved iterative adjustments, including the soldering and desoldering of various non-magnetic chip capacitors to achieve the desired resonant frequencies of 170 MHz, 300 MHz, and 650 MHz. This step was crucial for ensuring optimal performance of the coil in its intended application. The exact component values for each case, as well as the final tuning results, are provided in the preceding section of this document. Achieving accurate 50 Ω matching was particularly challenging and required careful adjustments to the passive components to ensure minimal reflection and maximum power transfer across the entire frequency range.

Throughout the fabrication and tuning process, careful attention was paid to maintaining the integrity of the coil’s design and ensuring that the final prototype met the desired performance criteria. The iterative process of component selection, tuning, and impedance matching is essential for optimizing birdcage coils in applications such as MRI and MR spectroscopy, where precise resonance and impedance characteristics are critical for achieving high-quality results.

The development process of a BC coil involves several stages:

(a) Define specifications: frequency, geometry, number of rungs, diameter, conductor type/size, and drive.

(b) Calculation of the passive component values required to achieve the desired resonant frequency and 50 Ω impedance [14]. This step ensures that the coil operates efficiently at the intended frequency and matches the system’s impedance.

(c) Construction of the BC prototype, which can be accomplished by either cutting the rungs and end rings from a copper sheet or utilizing a 3D printer. These fabrication techniques allow for precise coil geometries suited to the design specifications.

(d) Iterative tuning and optimization: Once the prototype is assembled, the process of adding or removing components (such as capacitors or inductors) is used to fine-tune the coil to achieve the required 50 Ω impedance matching, ensuring minimal signal reflection and efficient energy transfer.

(e) Performance characterization: S-parameters and quality factors.

(f) Image acquisition: Once the coil is tuned, images are obtained to assess the coil’s performance.

(g) Validation of the BC prototype: The final step involves validating the coil’s performance by comparing experimental results with theoretical predictions (Eq. (1)), confirming that the coil meets the design goals and performs as expected in practical applications.

**Fig. 3:**
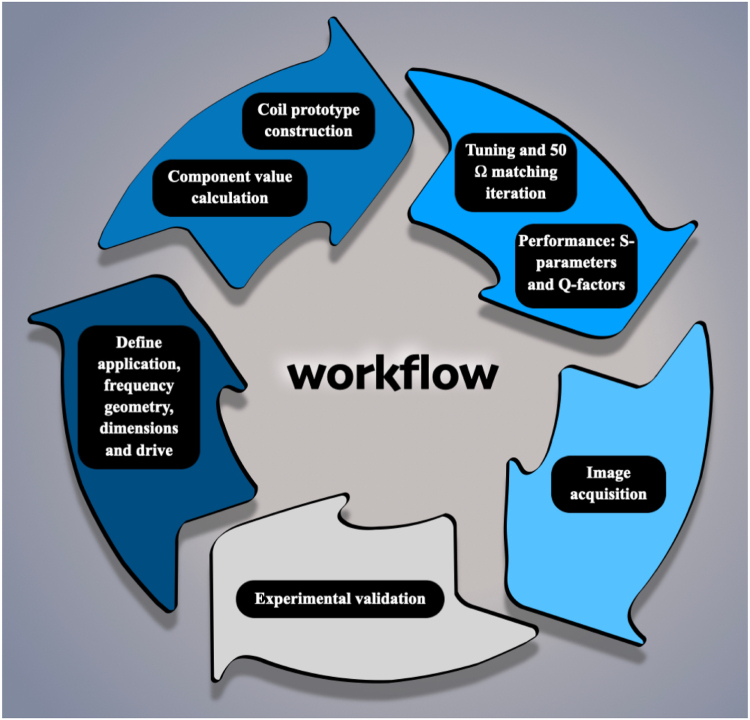
Schematic of BC coil development process: (a) Define specifications: frequency, geometry, number of rungs, diameter, conductor type/size, and drive. (b) Calculation of passive component values for resonant frequency and 50 Ω impedance [14]. (c) Construction of the prototype using copper sheet or 3D printing. (c) and (d) Iterative tuning for 50 Ω impedance matching. (e) Performance characterization: S-parameters and quality factors, (f) Image acquisition. (g) Validation through comparison of experimental and theoretical results.

Fig. 3 illustrates a schematic diagram outlining the complete process of constructing the in-house BC coils and validating the theoretical magnetic field expression.

## 6 Operation instructions

Before operating the birdcage (BC) coil within the MRI environment, users must follow strict safety protocols due to the high static magnetic field present in MR systems. All personnel must receive training from qualified staff before working near the scanner. It is critical to ensure that no ferromagnetic objects (e.g., tools, watches, smartphones) are brought near the magnet, as they may become dangerous projectiles. Additionally, individuals with pacemakers or medical implants are strictly prohibited from working within the MR room.

The BC resonator itself does not pose an additional safety risk, as its design exclusively utilizes non-ferromagnetic materials. The coil operates like any other MRI-specific RF coil and follows these operational steps:

1. **Connection:** Attach the BNC cable of the BC coil to the transmit/receive switch of the MRI system.
2. **Positioning:** Center the coil inside the MR bore, ensuring correct alignment with the scanner’s gradient system. The sample or subject is placed on the coil’s support tray.
3. **Tuning and Matching:** For optimal signal-to-noise ratio (SNR) and image quality, the coil must be properly tuned and impedance-matched.
  • Frequency tuning is performed via the two variable trimmers accessible through the coil?s endplate (see Fig. 1a). Initially, both trimmers should be turned fully up or down, then adjusted symmetrically to maintain field homogeneity.
  • Fine-tuning is achieved by incrementally adjusting one of the trimmers until the target resonance frequency is reached.
  • Impedance matching is done via a separate trimmer connected to the pickup loop. Adjust this trimmer to minimize reflected power and optimize energy transfer.
  • As tuning and matching parameters interact, these adjustments should be repeated iteratively until both frequency and impedance are properly set.
4. **Shimming:** Perform the necessary shimming process using the MRI system’s software to optimize the homogeneity of the static magnetic field.
5. **Image Acquisition:** Once the coil is tuned and shimmed, imaging sequences can be executed using the MRI software. The appropriate imaging parameters should be selected based on the experimental protocol.

These procedures ensure optimal performance and safety when using the BC coil in high-field MRI applications.

## 7 Validation and characterization

### 7.1 Imaging experiments

Phantom imaging experiments were conducted at various resonant frequencies using different acquisition parameters, pulse sequences, and MRI systems.

15.2 T: Using the coil prototype shown in Fig. 1.(d), imaging was performed with a saline solution-filled sphere (radius = 1 cm) utilizing a standard FLASH sequence. Acquisition parameters were as follows: TE/TR = 1.5 ms/120 ms, field of view (FOV) = 40 × 40 mm², matrix size = 256 × 256, slice thickness = 2 mm, flip angle = 45${}^{0}$ and NEX = 1. A transceiver quadrature birdcage coil (Bruker Co., Ettlingen, Germany; model RES 650 1H 059/035 QSN TR, inner diameter = 35 mm, outer diameter = 59 mm, length = 30 cm) was used for these experiments.

7 T: Fig. 1.(a) (In-house BC coil): A sphere (diameter = 2 cm) filled with a saline water solution was imaged on a research-dedicated 7 T/21 cm imager equipped with Direct Drive Technology (Agilent, Santa Clara, CA) using the in-house RF coil prototype. Phantom images were acquired using a standard gradient echo sequence with the following parameters: TR/TE = 1000/10 ms, FOV = 50 × 50 mm², matrix size = 256 × 256, slice thickness = 1 mm, NEX = 3, and flip angle = 20${}^{0}$. Fig. 1.(b–d) (In-house and vendor-provided BC coils): To validate the new coil design, imaging was performed using both the in-house BC coil and a vendor-provided birdcage coil (Bruker BioSpin MRI, GmbH, Ettlingen, Germany; model RF RES 300 1H 075/040 QSN TR, model no.: 1PT13161V3, serial no.: S0121, REV/VEC: 2P01.05). Imaging experiments were conducted on a 7T/30 cm Bruker imager using a standard gradient echo sequence.

4 T: Using the coil prototype shown in Fig. 1.(e), imaging was conducted in a whole-body superconducting magnet interfaced with an INOVA console (Varian, Inc., Palo Alto, CA, USA) and SONATA gradients (Siemens). A spherical phantom (radius = 9 cm) was filled with distilled water containing a solution of creatine (50 mM), N-acetyl aspartate (12.5 mM), choline (3.0 mM), myo-inositol (7.5 mM), and glutamate (12.5 mM). Phantom images were acquired using the coil prototype and a spin-echo sequence with the following parameters: TR/TE = 3000/130 ms, FOV = 160 × 160 mm², matrix size = 256 × 256, slice thickness = 5 mm, and NEX = 1.

The summarized data from these experiments is presented in Table 1 (see Table 3).

**Fig. 4:**
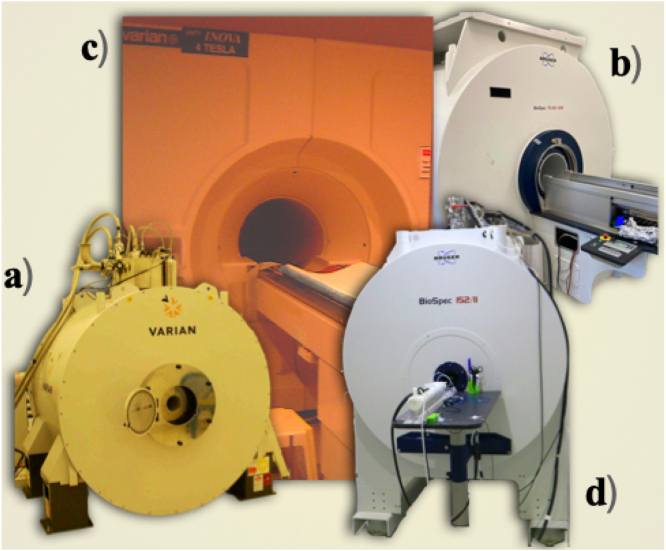
MRI systems used to experimentally test the in-house and vendor-provided birdcage (BC) coils at different resonant frequencies: (a) a 7 T/12 cm imager from Agilent (Varian, Santa Clara, CA) with actively shielded gradients (400 mT/m maximum strength); (b) a 7 T/30 cm imager from Bruker BioSpin (GmbH, Ettlingen, Germany) with high-performance gradients (1000 mT/m strength, 9000 T/m/s slew rate); (c) a 4 T/60 cm imager using an INOVA (Varian, Santa Clara, CA) console with Siemens SONATA gradients (70 mT/m strength, 200 T/m/s slew rate); and (d) a 15.2 T/11 cm imager from Bruker BioSpin with an actively shielded gradient system (1000 mT/m strength, 9000 T/m/s slew rate).

Fig. 4 provides photographs of the three MR imagers used to acquire the phantom images detailed above. These MRI systems were essential for evaluating the performance of the birdcage coil prototypes across different field strengths and imaging setups.

**Table 3:** Summary of birdcage coil configurations, imaging protocols, and performance metrics.

Reference	$B_{0}$ [T]	BC type/drive	Sequence	Rungs/length/diameter [mm]	$Q_{l}/Q_{u}$	SNR/NF
[8]	7	High-pass/quadrature	Gradient echo	16/105/50	35/39	84/1.35
[7]	7	High-pass/quadrature	Spin echo	4/64/40	96/125	24.34/1.4
[10]	7	Transceiver mode/quadrature	Spin echo	6/80/50	Not available	37.77/1.21
[6]	7	High-pass/quadrature	Gradient echo	4/113/66	43/105	76.54/2.6
[11]	4	Pass-band/quadrature	Spin echo	4/190/180	18.888/20.035	41.14/1.58
[12]	15.2	Transceiver mode/quadrature	Gradient echo	6/30/60	Not available	28.3/1.35

**Fig. 5:**
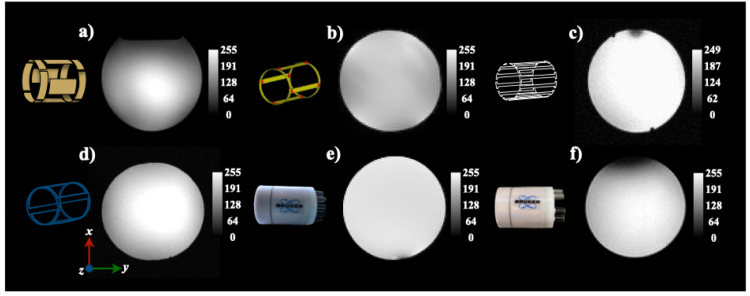
Phantom images acquired using the in-house and vendor-provided birdcage (BC) coils: (a) 4 T, (b)–(e) 7 T, and (f) 15.2 T, with the MRI systems described in Fig. 4.

Fig. 5 presents phantom images acquired using both in-house and vendor-provided BC coils in combination with different MR imagers. Overall, the images exhibit excellent quality, with clear and distinct representations of the phantom. However, some susceptibility artifacts are observed in Fig. 4.(c), (e), and (f). These artifacts are typically associated with issues related to the construction and handling of the coil prototypes, which can lead to variations in the magnetic field distribution. Specifically, the artifact seen in Fig. 4.(f) is caused by the proximity of a coaxial cable to the BC coil, which introduces local field distortions. Polylactic Acid (PLA) is unsuitable for constructing birdcage coils at high frequencies due to its significant magnetic susceptibility. The inherent diamagnetism of PLA creates a substantial susceptibility mismatch with air and tissue, distorting the scanner’s homogeneous magnetic field. This distortion causes severe image artifacts, including geometric warping and signal loss, while also detuning the resonator and degrading RF field homogeneity. Given that these effects intensify with field strength, high-field applications require alternative materials such as ABS, polycarbonate, or Delrin, which have susceptibilities closer to air, to ensure image fidelity and spectral quality.

Despite these artifacts, it is important to emphasize that they did not have a significant impact on the overall image quality or the ability to interpret the data. The image resolution and clarity remained high across all cases, suggesting that the coil designs, while still prototypes, perform well even in the presence of minor imperfections. Furthermore, the results demonstrate the robustness of the imaging system, as good image quality was maintained even when different BC coils were used with various MR imagers. This highlights the versatility and reliability of the approach, making it suitable for a wide range of applications, even in cases where coil construction may not be entirely optimized.

In summary, while susceptibility artifacts were present, their impact on the final image quality was minimal, and the overall performance of the BC coils across different MR systems was consistently strong, reinforcing the feasibility of using these coils in practical imaging scenarios.

### 7.2 Theoretical $B_{1}$ validation

Bidinosti et al. [15] developed an expression for the magnetic field, $B_{1}$, produced by an RF volume coil within a spherical volume, extending the theoretical framework established by London [16]. Their formulation accommodates a sphere with arbitrary permeability μ, permittivity ɛ, and electrical conductivity σ. In this section, we derive and present the key equation for $B_{1}$ inside the sphere (Eq. (7)), followed by a discussion of how this theoretical profile is compared against experimental data.

#### 7.2.1 Sphere model in a uniform RF field

According to London [16], consider a sphere of radius a, located at the origin, with permeability μ, permittivity ɛ, and electrical conductivity σ. This sphere is placed in a uniform applied RF field

$$B_{1}\left(t\right)\;=\;B_{1}\;e^{-i\omega t}\;\overset{ˆ}{z}.$$

We begin our analysis with the Maxwell-Ampere equation:

(1) $$\nabla \times B\;=\;\mu_{0}\;J,$$

where J is the total current density, and $\mu_{0}$ is the permeability of free space.

Under spherical symmetry at radio frequencies, we consider only the azimuthal component of the current density:

(2) $$J_{\varphi }=f\left(r\right)\sin\theta a_{\psi },$$

where f(r) is a radial function describing the spatial dependence of the azimuthal current within the sphere, and θ is the polar angle. This function f(r) satisfies:

(3) $$f^{\prime \prime }\left(r\right)+\frac{2}{r}f^{\prime }\left(r\right)+\left(k^{2}-\frac{2}{r^{2}}\right)f\left(r\right)=0$$

where $k=\sqrt{\mu \omega \left(ɛ\omega +i\sigma \right)}$ is the complex propagation constant. The general solution to Eq. (3) is:

(4) $$f\left(r\right)=A\;j_{1}\left(kr\right)+B\;y_{1}\left(kr\right),$$

where $j_{n}$ and $y_{n}$ are spherical Bessel functions of the first and second kind, respectively. To avoid divergence at r=0, we set B=0, yielding

(5) $$f\left(r\right)=A\;j_{1}\left(kr\right).$$

Hence, the azimuthal current density becomes:

(6) $$J_{\psi }\left(r,\theta \right)=f\left(r\right)\;\sin\theta =A\;j_{1}\left(kr\right)\;\sin\theta .$$

#### 7.2.2 Boundary conditions and final form of $B_{1}$

Applying the usual boundary conditions for continuity of tangential components at the sphere’s surface r=a leads to a solution for the constant A. A more compact approach shows that, inside a dielectric sphere subjected to a uniform RF field at angular frequency ω, the magnetic field $B_{1}\left(r,\theta \right)$ can ultimately be written in the form:

(7) $$B_{1}\left(r,\theta \right)=\frac{2\;J_{\psi }}{3\;i\;\omega \;\sigma }\;\frac{k\;j_{0}\left(ka\right)}{j_{1}\left(kr\right)\;\sin\theta },$$

where

$$J_{\psi }\left(r,\theta \right)=\frac{3\;i\;\omega \;\sigma \;B_{1}}{2}\;\frac{\;j_{1}\left(kr\right)}{\;k\;j_{0}\left(ka\right)}\;\sin\theta .$$

Eq. (7) describes the $B_{1}$ magnetic field generated within a spherical sample by an external RF coil. The field distribution is governed by the complex propagation constant $k=\sqrt{\mu \omega \left(\epsilon \omega +i\sigma \right)}$, which encapsulates the electromagnetic response of the sample (through μ, ϵ, and σ) at the operating frequency ω. The spatial variation is described by spherical Bessel functions $j_{0}$ and $j_{1}$, with r, θ, and a defining the geometry.

#### 7.2.3 Analytical formulation and computation procedure

Although both $B_{1}$ and $J_{\psi }$ appear in Eq. (7), there is no circular dependence between them. The derivation proceeds by first solving Eq. (3) for the azimuthal current density $J_{\psi }\left(r,\theta \right)$ using the boundary condition at r=a, where the tangential component of the magnetic field is continuous with the applied field $B_{1}^{\mathrm{ext}}$. This boundary field acts as a known excitation, determining the amplitude of $J_{\psi }$ inside the sphere. Once $J_{\psi }$ is obtained, it is substituted into Maxwell–Ampère’s law to yield the internal distribution $B_{1}\left(r,\theta \right)$. In practical computations, $B_{1}^{\mathrm{ext}}$ is defined as the incident RF field amplitude at the sphere surface (e.g., $B_{1}^{\mathrm{ext}}=1$ A/m for normalization), and Eq. (7) then provides the full spatial profile of the internal field. Thus, $J_{\psi }$ is a dependent quantity derived from $B_{1}^{\mathrm{ext}}$, not an independent unknown.

A key point is that this solution for $B_{1}$ is fundamental. The current density $J_{\psi }$ is a consequence of the boundary conditions applied to this field, not an independent quantity. Therefore, $B_{1}$ and $J_{\psi }$ cannot be solved separately, preventing a potential misconception. From an engineering perspective, this model is highly practical. By inputting measurable geometric and material properties, one can predict the $B_{1}$ field for a birdcage-like coil. This makes Eq. (7) a vital quantitative tool for optimizing coil performance at ultra-high fields, enabling designers to enhance field uniformity and maximize SNR. Thus, replicating the result from Bidinosti et al. [15], this expression provides the theoretical basis for both validating $B_{1}$ mapping experiments and providing an intuitive framework for the initial coil design process.

#### 7.2.4 Practical use in birdcage (BC) coil design

When designing an RF birdcage coil, one can leverage Eq. (7) to predict the coil’s sensitivity ($B_{1}$ uniformity) inside a spherical region, even at ultra-high fields. In practice, coil dimensions (length l, diameter d, number of segments N, etc.) appear in the factor $B_{\mathrm{center}}$, which is the on-axis field amplitude. Detailed formulas for $B_{\mathrm{center}}$ without shielding are found in Refs. [15], [16] and can be used to tailor coil geometry for optimal uniformity.

#### 7.2.5 Limitations and applicability of the model

**Fig. 6:**
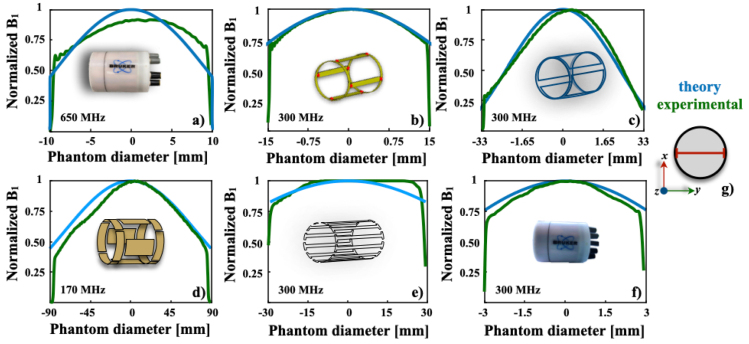
Comparison of uniformity plots generated from the phantom image data in Fig. 5 and Eq. (7) at magnetic field strengths of 4 T (d), 7 T (b, c, e, f), and 15.2 T (a). All profiles were acquired along the red line shown in (g).

The derivation of Eq. (7) relies on a simplified model of an ideal spherical sample with homogeneous properties. In reality, dielectric effects and conductivity losses ($\epsilon^{\prime \prime }$, σ) at ultra-high fields cause significant $B_{1}$ inhomogeneities that are not fully captured, leading to discrepancies between theory and experiment, particularly near the sample boundary (Fig. 6). Therefore, while the analytical model shows good correlation near the coil center, its accuracy is limited for realistic, heterogeneous samples. It should be viewed as a first-pass design tool: excellent for scoping coil geometry and understanding fundamental behavior quickly. For final, accurate $B_{1}$ field predictions, especially in large or conductive samples, this model must be supplemented with advanced techniques like full-wave electromagnetic simulations.

#### 7.2.6 Comparison with experimental data

Using Eq. (7), we generated theoretical $B_{1}$ (or uniformity) profiles for each birdcage (BC) coil tested. Fig. 6 (below) compares these theoretical profiles with the uniformity profiles extracted from our experimental image data. All profiles were taken along the same reference line (indicated in Fig. 6(g)), ensuring a direct comparison. The close agreement between the calculated and measured curves confirms that the BC coils perform as anticipated in terms of $B_{1}$ uniformity.

As illustrated in Fig. 6, the experimental data demonstrate a strong correlation with the theoretical predictions of Eq. (7). This agreement is qualitatively evident in the high fidelity with which the model reproduces the experimental $B_{1}$ field distribution. Specifically, the characteristic saddle-shaped profile of the birdcage coil is clearly replicated, showing excellent concordance in key features: the central region of high field uniformity, the predictable decline in field strength along the *z*-axis towards the coil’s end-rings, and the consistent azimuthal symmetry around the central conductor elements. Notably, the model accurately captures the subtler field modulations at the boundaries between the coil’s rungs and end-rings, areas where complex fringe fields and capacitive effects often lead to discrepancies in simpler models. The absence of any significant localized hotspots or nulls in the experimental data relative to the prediction further underscores the model’s robustness.

This comprehensive qualitative agreement provides a compelling visual validation of both the coil’s practical construction and the underlying theoretical model, which successfully incorporates the essential electromagnetic interactions of the resonator. This finding powerfully supports a streamlined design approach where coil dimensions and tuning can be optimized computationally prior to physical construction, thereby circumventing the traditional, resource-intensive cycle of iterative simulations and empirical adjustments. By offering a systematic and accurate framework for predicting performance, this method is invaluable for evaluating both commercial and custom birdcage coils. It empowers researchers and engineers to develop more efficient, application-specific prototypes for ultra-high-field MRI, significantly accelerating the transition from concept to a validated, functional coil. Consequently, the theoretical and experimental comparisons confirm that the proposed framework offers a physically accurate and predictive description of birdcage coil behavior.

#### 7.2.7 Coil performance: SNR and Noise Figure

In essence, while SNR is the final measure of image quality that the radiologist cares about, the Noise Figure is a fundamental engineering specification that determines the scanner’s ability to achieve that SNR. You measure the SNR to see the final result, but you optimize and control the Noise Figure to ensure the hardware is capable of delivering the best possible result. This relationship becomes clear when we define what constitutes good image quality with specific SNR values. It’s important to note that there is no single universal good SNR, as it is highly dependent on the anatomical region, spatial resolution, and clinical question. The birdcage coils in this study demonstrate consistently high SNR and low Noise Figure across the operational frequencies, establishing a strong foundation for developing next-generation, high-performance coils. The key results are summarized in Table 1.

## 8 Limitations

Although our BC coil prototypes exhibit robust performance, a few limitations should be noted:

• **Construction-induced artifacts:** Minor artifacts can arise from small irregularities in coil construction (e.g., unoptimized solder joints, uneven copper strips) or the proximity of coaxial cables, leading to local distortions in the magnetic field.
• **High-field tuning complexity:** At very high frequencies or for larger sample volumes, precise tuning and matching can be challenging and require repeated iterative adjustments or additional hardware (like cable traps).
• **Scope of phantom testing:** Our tests focused on spherical phantom images rather than *in vivo* imaging, so further validation may be needed for clinical or biological studies where tissue conductivity and susceptibility may vary.

## 9 Conclusions

We have demonstrated an analytical method to validate the magnetic field generated by birdcage coils at multiple field strengths (4 T, 7 T, and 15.2 T). The strong agreement between our theoretical predictions and phantom imaging data confirms that this approach reliably captures the key physics governing $B_{1}$ distribution. Compared to fully numerical simulations, our streamlined model offers a faster, cost-effective way to guide coil design. By integrating basic spherical Bessel expansions with practical build-and-test protocols, researchers can more easily tune birdcage coils to specific applications—from preclinical rodent studies to larger-scale systems. These results underscore the potential of our method to expedite RF coil prototyping and refinement, even at ultra-high fields.

## CRediT authorship contribution statement

**A. Villarreal:** Investigation, Resources, Software, Visualization. **J. Lazovic:** Formal analysis, Methodology, Validation, Writing – review & editing. **S.E. Solis-Najera:** Investigation, Methodology, Supervision, Validation, Visualization, Writing – review & editing. **R. Martin:** Formal analysis, Investigation, Validation, Visualization, Writing – original draft. **R. Ruiz:** Data curation, Software, Visualization. **L. Medina:** Conceptualization, Methodology, Visualization. **A.O. Rodriguez:** Conceptualization, Funding acquisition, Investigation, Methodology, Supervision, Writing – original draft, Writing – review & editing.

## Declaration of competing interest

The authors declare that we have no competing interests regarding the research and publication of this paper. All potential conflicts of interest have been disclosed in accordance with the relevant guidelines.
